# The XRCC1 Arg194Trp polymorphism is not a risk factor for glioma: A meta-analysis involving 1,440 cases and 2,562 controls

**DOI:** 10.3892/etm.2012.716

**Published:** 2012-09-18

**Authors:** LIANG ZHANG, YAN WANG, ZHIQUN QIU, JIAOHUA LUO, ZIYUAN ZHOU, WEIQUN SHU

**Affiliations:** 1 Department of Environmental Hygiene, College of Preventive Medicine, Third Military Medical University, Chongqing 400038;; 2 Institute of Respiratory Diseases, Xinqiao Hospital, Third Military Medical University, Chongqing 400037, P.R. China

**Keywords:** X-ray repair cross-complementing gene 1 Arg194Trp, glioma, malignancy, susceptibility, meta-analysis, polymorphism

## Abstract

Previous reports have indicated that the X-ray repair cross-complementing gene 1 (XRCC1) Arg194Trp polymorphism may be a risk factor for several types of cancer. Published studies on the association of XRCC1 Arg194Trp polymorphisms with glioma risk have yeilded conflicting results. The present study aimed to obtain a more precise estimation of this association. Meta-analyses assessing the association of the XRCC1 Arg194Trp variation with glioma were conducted and subgroup analyses based on ethnicity and source of controls were also performed. Eligible studies were identified during the period before May 2012. A total of four case-control studies comprising 1,440 cases and 2,562 controls were finally selected for analysis. The overall data failed to indicate a significant association of the XRCC1 Arg194Trp polymorphism with glioma risk [Trp vs. Arg: odds ratio (OR) = 1.01, 95% confidence interval (95% CI) = 0.77–1.33; Trp/Trp vs. Arg/Arg: OR = 1.56, 95% CI = 0.96–2.54; dominant model: OR = 0.98, 95% CI = 0.74–1.31; recessive model: OR = 1.48, 95% CI = 0.92–2.38]. Similarly, in the subgroup analysis based on ethnicity and source of controls, no associations were observed. In conclusion, the results of the present study failed to suggest an association between the XRCC1 Arg194Trp polymorphism and glioma risk. Further large and well-designed studies are required to confirm this conclusion.

## Introduction

Glioma is the most common type of primary brain tumor in adults. The general outcomes for patients are poor, particularly for older patients. The etiology of glioma is unclear. Evidence suggests that exposure to radiation may be a significant risk factor for glioma, which may explain a small proportion of glioma since this exposure is generally rare (1). However, only a minority of individuals exposed to radiation eventually develop glioma, indicating that host genetic factors may play a critical role in the carcinogenesis of glioma (2).

Radiation exposure may cause DNA damage and cell injury. The consequences to the cells may be disastrous, ranging from single gene mutations to massive chromosomal breakdown and rearrangements. The instabilities may result in severe human disorders, including cancer (3). The repair of various types of DNA damage is vital for the maintenance of genomic stability and cell survival. Base excision repair pathways are critical in this process. X-ray repair cross-complementing gene 1 (XRCC1) is one of the most important DNA repair genes that plays a key role in the process of base excision repair. The XRCC1 gene is located on chromosome 19q13.2–13.3 and is 33 kb in length, comprising 17 exons and encoding a 70-kDa protein (4). A widely studied XRCC1 single nucleotide polymorphism at codon 194, with a Arg to Trp alteration (rs1799782), may have a diminished capacity to remove DNA adducts and oxidized DNA damage (5). Hence, the Arg194Trp variation has been associated with cancer susceptibility.

Published data on the association of the XRCC1 Arg194Trp polymorphism with glioma have yielded controversial results. In the present study, we carried out a quantitative meta-analysis that increased statistical power to derive a more precise estimation of this association.

## Materials and methods

### Literature search strategy

We performed a search of the Medline, Embase, Ovid, Sciencedirect and Chinese National Knowledge Infrastructure (CNKI) databases without a language limitation, including all studies published until May 2012, with a combination of the following keywords: XRCC1, Arg194Trp, glioma, brain, neoplasm, cancer, variation and polymorphism. All searched studies were retrieved and the bibliographies were reviewed for other relevant publications. Review articles and bibliographies of other relevant studies identified were searched manually to identify additional eligible studies.

### Inclusion criteria

The following criteria were used for the literature selection: i) studies should report the association of the XRCC1 Arg194Trp polymorphism with glioma risk; ii) studies are observational studies (case-control or cohort); iii) studies should provide the sample size, odds ratios (ORs) and 95% confidence intervals (CIs), the genetic distribution or the information to infer the results. After rigorous searching, we reviewed all studies in accordance with the criteria defined above for further analysis.

### Data extraction

Data were carefully extracted from all eligible publications independently by two of the authors according to the inclusion criteria mentioned above. For conflicting evaluations, an agreement was reached following a discussion. If a consensus could not be reached, another author was consulted to resolve the dispute and then a final decision was made by a majority of the votes. Extracted information was entered into a database.

### Statistical analysis

The OR of the XRCC1 Arg194Trp polymorphism and glioma risk was estimated for each study. The pooled ORs were assessed for the genetic comparisons of allelic contrast (Trp vs. Arg), homozygote comparison (Trp/Trp vs. Arg/Arg), dominant model (Trp/Trp + Trp/Arg vs. Arg/Arg) and recessive model (Trp/Trp vs. Trp/Arg + Arg/Arg). For the detection of any possible sample size biases, the OR and 95% CI for each study was plotted against the number of participants. A Chi-square based Q statistic test was performed to assess heterogeneity. If the result of the heterogeneity test was P>0.1, ORs were pooled according to the fixed-effects model (Mantel-Haenszel); otherwise, the random-effects model (DerSimonian and Laird) was used. The significance of the pooled ORs was determined by the Z-test. The Hardy-Weinberg equilibrium (HWE) was assessed by Fisher’s exact test.

Publication bias was assessed by visual inspection of funnel plots (6), in which the standard error of log (OR) of each study was plotted against its log (OR). An asymmetric plot indicates a possible publication bias. The symmetry of the funnel plot was further evaluated by Egger’s linear regression test (7). Statistical analysis was undertaken using the program STATA

## Results

### Study characteristics

**Figure 1:**
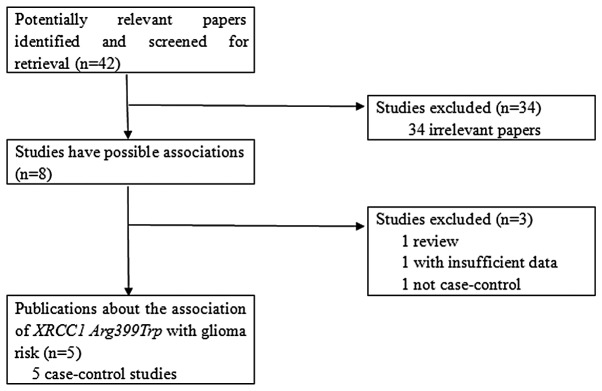
Flow diagram of included and excluded studies.

Relevant publications were retrieved and screened. As shown in Fig. 1, a total of 42 publications were identified, of which 34 irrelevant studies were excluded. Thus, eight publications were eligible in preliminary stages, of which one review article (8) and one non-case-control study (9) were discarded. Subsequently, one study that did not provide the detailed distributions of the genotypes (10) was excluded. Five case-control studies were included for data extraction and analysis (11–15). However, a study conducted by Custodio *et al* (13) was excluded since it contributed to evident heterogeneity for the overall data under the four genetic models. Lasty, four case-control studies were selected for analysis (11,12,14,15).

All the selected publications were written in English. The relevant information is listed in Table I, including the first author and the number and characteristics of cases and controls for each study as well as other relevant information. There were two groups of Asians (14,15) and two of Caucasians (11,12) in the present meta-analysis.

The distributions of the XRCC1 Arg194Trp genotypes as well as the genotyping methods of the included studies are presented in Table II. The genetic distributions of the control groups in all studies were consistent with the HWE, with the exception of one study (14).

### Test of heterogeneity

As shown in Table III, we analyzed the heterogeneities for the four genetic comparisons. Evident heterogeneities for the overall data were present in the allelic contrast (P=0.030, Q-test) and dominant model (P=0.061 for Q-test), with the exception of the homozygote comparison (P=0.561, Q-test) and recessive model (P=0.598, Q-test). Additionally, the I-square value is another index for a heterogeneity test (16), with value less than 25% indicating low, 25% to 50% indicating moderate, and greater than 50% indicating high heterogeneity. The I-square values were 66.6 and 59.2% for the overall data of the allelic contrast and dominant model, respectively, suggesting statistically significant heterogeneity between the studies; therefore, the random-effects models were utilized. For the homozygote comparison and recessive model, the I-square values were both 0.0%, indicating an absence of the heterogeneity. Thus, the fixed-effects model was used in these two models.

Notably, when the overall data were divided for subgroup analysis, we observed a loss of heterogeneity in the subgroups in the allelic contrast and dominant models.

### Meta-analysis results

The main results of the meta-analysis are listed in Table III. For the overall data including 1,440 cases and 2,562 controls, significant associations of the XRCC1 Arg194Trp polymorphism with glioma risk were not identified in the four genetic models (allele contrast: OR = 1.01, 95% CI = 0.77–1.33; homozygote comparison: OR = 1.56, 95% CI = 0.96–2.54; dominant model: OR = 0.98, 95% CI = 0.74–1.31; recessive model: OR = 1.48, 95% CI = 0.92–2.38), indicating that the XRCC1 Arg194Trp polymorphism may not have a correlation with glioma risk.

**Figure 2:**
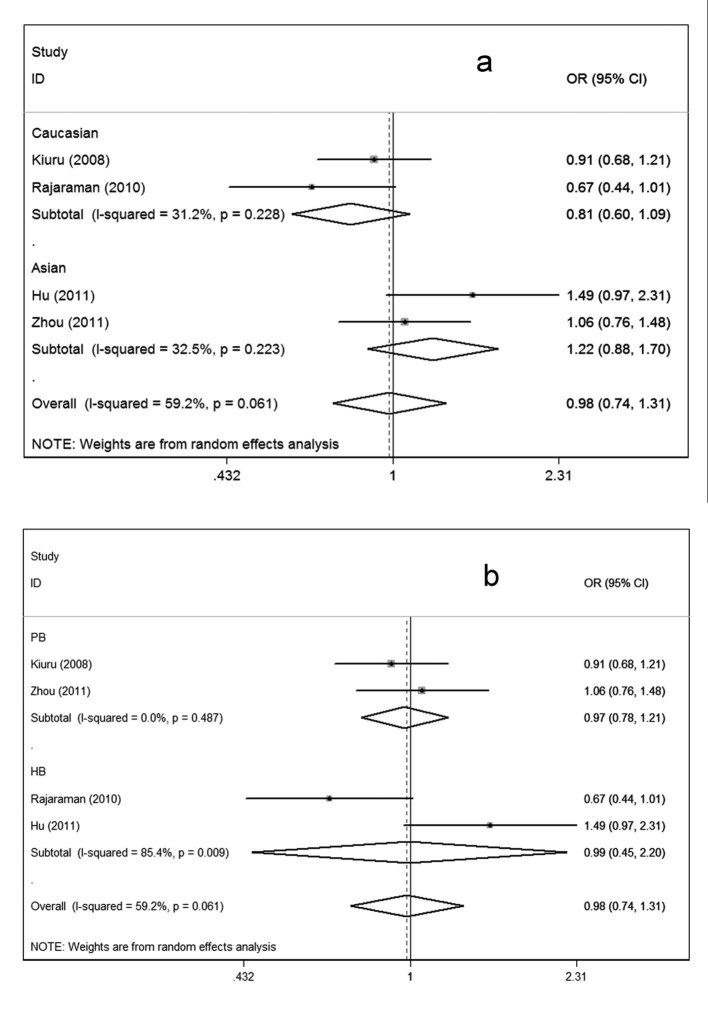
Meta-analysis for the association of glioma risk with the X-ray repair cross-complementing gene 1 (XRCC1) Arg194Trp polymorphism. (Trp/Trp + Trp/Arg vs. Arg/Arg). (A) Stratified by ethnicity. (B) Stratified by source of controls. OR, odds ratio; CI, confidence interval.

Considering the possible effects of ethnic variation and selection of controls on the results (Fig. 2), we conducted subgroup analyses. In the subgroup analysis according to ethnicity, no associations were observed in either the Asian or Caucasian subgroup (Fig. 2a). Similarly, in the subgroup analysis by source of controls, significant associations could be observed in neither the population-based subgroups nor the hospital-based subgroups under the four genetic comparisons (Fig. 2b).

### Sensitivity analysis

To test the stability of the overall results, we carried out one-way sensitivity analysis (17). The statistical significance of the results was not changed when any single study was omitted (data not shown), indicating the robustness of the results.

### Bias diagnostics

**Figure 3:**
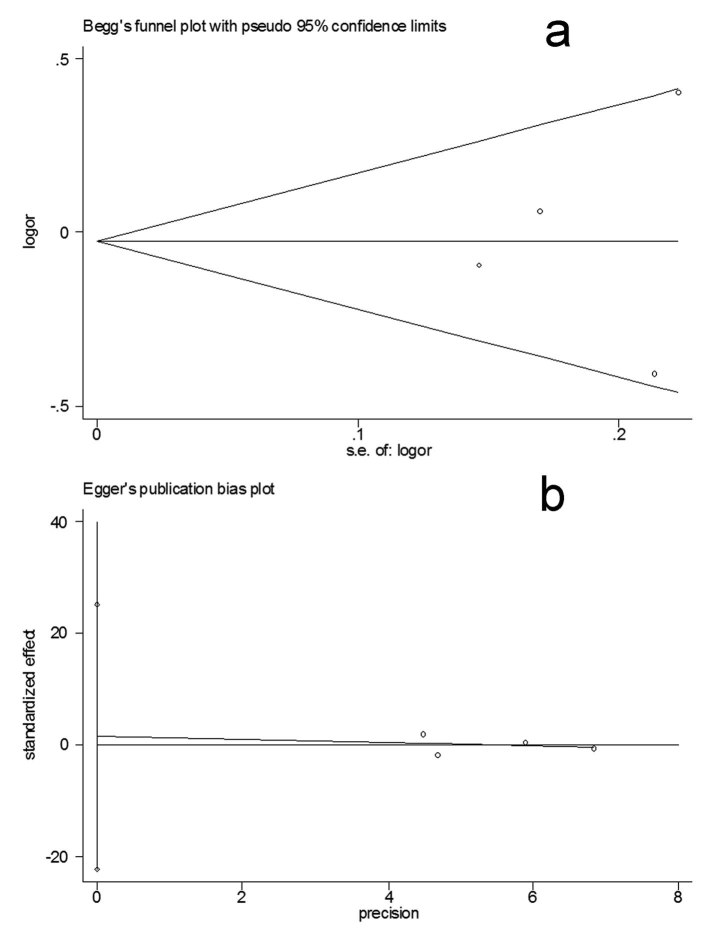
Publication bias test for the overall data (Trp/Trp + Trp/Arg vs. Arg/Arg). (A) Funnel plot. (B) Egger’s linear regression test.

Funnel plots were created for assessment of possible publication biases (Fig. 3a). Then, Egger’s linear regression tests were used to assess the symmetries of the plots. The funnel plots appeared to be symmetrical for the overall data indicated by the Egger’s tests (allelic contrast: t=−0.33, P>0.05, homozygote comparison: t=−0.24, P>0.05; dominant model: t=0.26, P>0.05; recessive model: t=−0.13, P>0.05; Fig. 3b), suggesting that the publication biases were not evident.

## Discussion

The overall data obtained failed to show a significant association between the XRCC1 Arg194Trp polymorphism and glioma risk. Similar results were demonstrated in the subgroup analysis based on ethnicity and source of controls.

Previous meta-analyses have focused on the association between the XRCC1 Arg194Trp polymorphism and several cancer risks and have generated conflicting results. The XRCC1 Arg194Trp variation has been demonstrated to increase risk of lung and gastric cancer (18,19). However, meta-analyses regarding skin and esophageal cancer failed to reveal such associations (20,21). Therefore, the XRCC1 Arg194Trp polymorphism may exert different effects on different cancers.

In the subgroup analysis based on ethnicity, no significant associations could be observed in the Asian or Caucasian subgroup. Evidence indicates that ethnic-specific variation, different health care and socioeconomic class might exert an effect on the incidence of glioma (22). The results demonstrated that the potential effects of ethnic variations on glioma were not evident. Notably, the results should be interpreted with care since the limited number of the included studies containing small sample sizes may result in insufficient statistical power to evaluate a small effect. Therefore, future investigations regarding different ethnicities with large sample sizes are required to clarify this issue.

When the data were stratified based on the source of controls, no association was observed in either the population-based or hospital-based subgroup. Since hospital-based controls may not be representative of the general population, a selection bias may exist. However, the data of the present meta-analysis suggest that the potential influence of the selection bias on the overall results was not evident. However, use of proper control participants with rigorous matching criteria and large sample sizes in future studies is important to reduce such possible selection bias.

In the present meta-analysis, evident between-study heterogeneities for overall data were observed in the allelic contrast and dominant models. However, when the subgroup analyses were conducted, we found that the heterogeneities were removed in the subgroups regarding Caucasian, Asian and population-based controls. However, the heterogeneity was still present in the hospital-based subgroup. Therefore, the heterogeneities might be multifactorial. In addition to ethnicity and source of controls, other factors including age, pathology grade and life styles may also contribute to the heterogeneity.

Several limitations should be addressed. First, in this meta-analysis, the primary articles only provided data regarding Caucasians, Asians and mixed races. Other ethnicities such as African should be investigated in future studies. Second, subgroup analyses based on age, gender, histological types, radiation exposure and other factors have not been performed in the present study since sufficient relevant data were not available in the primary literature. Third, only studies written in English and several other languages indexed by the common databases were searched. Thus, a selection bias might exist. Therefore, the results should be interpreted with caution. However, the sensitivity analysis and publication bias analysis indicated the stability and credibility of the present meta-analysis.

In conclusion, the results of the present meta-analysis failed to suggest an association of the XRCC1 Arg194Trp polymorphism with glioma risk. Further investigations with larger sample sizes and rigorous matching criteria in view of confounding factors are required to confirm the associations.

## Figures and Tables

**Table I:** Characteristics of studies included in the meta-analysis.

Authors/(Refs.)	Publication year	No. of cases (male/female)	No. of controls (male/female)	Type of controls	Median (or mean)age (range) in years (cases/controls)	Ethnic decent	Country
Kiuru *et al* (11)	2008	426 (259/167)	1560 (705/855)	Healthy controls (age-, gender-, geographical area-matched; PB)	48.2 (NA)/63 (NA)	Caucasian	Four countries in Europe
Rajaraman *et al* (12)	2010	362 (198/164)	495 (228/267)	Non-cancer controls (age-, race-, gender-, hospital-, residence-matched; HB)	51.2 (18–90)/49.2 (18–90)	Caucasian	USA
Hu *et al* (14)	2011	127 (87/40)	249 (166/83)	Non-cancer controls (age-, gender-matched; HB)	49.5 (NA)/48.9 (NA)	Asian	China
Zhou *et al* (15)	2011	271 (168/103)	289 (180/109)	Healthy controls (age-matched; PB)	47.8 (NA)/46.9 (NA)	Asian	China

NA, not available; PB, population-based; HB, hospital-based.

**Table II:** Distribution of the XRCC1 Arg194Trp genotypes among glioma cases and controls included in the meta-analysis.

			Cases			Controls			
Authors/(Refs.)	Year	Genotyping method	Trp/Trp	Arg/Trp	Arg/Arg	Trp/Trp	Arg/Trp	Arg/Arg	HWE (control)
Kiuru *et al* (11)	2008	PCR-RFLP	3	71	626	2	177	1,377	Yes
Rajaraman *et al* (12)	2010	TaqMan	0	38	304	1	73	394	Yes
Hu *et al* (14)	2011	PCR-CTPP	18	38	71	22	64	163	No
Zhou *et al* (15)	2011	TaqMan	14	112	145	13	117	159	Yes

PCR-RFLP, polymerase chain reaction-restriction fragment length polymorphism; PCR-CTPP, polymerase chain reaction with confronting two-pair primers; HWE, Hardy-Weinburg equilibrium.

**Table III:** Main results of the pooled data from the meta-analysis.

		Trp allele vs. Arg allele			Trp/Trp vs. Arg/Arg			(Trp/Trp + Trp/Arg) vs. Arg/Arg			Trp/Trp vs. (Trp/Arg + Arg/Arg)		
Analysis	No. (cases/controls)	OR (95% CI)	P	P (Q-test)	OR (95% CI)	P	P (Q-test)	OR (95% CI)	P	P (Q-test)	OR (95% CI)	P	P (Q-test)
Total	1440/2562	1.01 (0.77–1.33)	0.933	0.030	1.56 (0.96–2.54)	0.073	0.561	0.98 (0.74–1.31)	0.909	0.061	1.48 (0.92–2.38)	0.107	0.598
Ethnicity													
Caucasian	1042/2024	0.83 (0.60–1.14)	0.240	0.180	1.83 (0.43–7.88)	0.415	0.274	0.81 (0.60–1.09)	0.160	0.228	1.88 (0.44–8.10)	0.395	0.284
Asian	398/538	1.23 (0.89–1.71)	0.214	0.131	1.53 (0.91–2.57)	0.107	0.383	1.22 (0.88–1.70)	0.231	0.223	1.44 (0.87–2.38)	0.157	0.456
Source of controls													
PB	971/1845	1.00 (0.83–1.21)	0.998	0.551	1.39 (0.68–2.85)	0.368	0.303	0.97 (0.78–1.21)	0.797	0.487	1.36 (0.67–2.76)	0.391	0.286
HB	469/717	1.01 (0.47–2.18)	0.983	0.004	1.73 (0.89–3.35)	0.104	0.378	0.99 (0.45–2.20)	0.990	0.009	1.59 (0.83–3.03)	0.158	0.428

P, P-value; OR, odds ratio; CI, confidence interval; PB, population-based; HB, hospital-based.
